# Novel insights into antioxidant status, gene expression, and immunohistochemistry in an animal model infected with camel-derived *Trypanosoma evansi* and *Theileria annulata*

**DOI:** 10.1186/s13071-024-06564-3

**Published:** 2024-11-18

**Authors:** Reem M. Ramadan, Alaa F. Bakr, Esraa Fouad, Faten F. Mohammed, Azza M. Abdel-Wahab, Sahar Z. Abdel-Maogood, Mohamed M. El-Bahy, Mai A. Salem

**Affiliations:** 1 Department of Parasitology, Faculty of Veterinary Medicine, Cairo University, Giza, 1221 Egypt; 2 Department of Pathology, Faculty of Veterinary Medicine, Cairo University, Giza, 1221 Egypt; 3 The Central Laboratory for Evaluation of Veterinary Biologics (CLEVB), Agriculture Research Centre (ARC), Cairo, Egypt; 4 Department of Pathology, College of Veterinary Medicine, King Faisal University, 31982 Al-Ahsa, Saudi Arabia

**Keywords:** *Trypanosoma evansi*, *Theileria annulata*, Camels, Gene expression, Stress markers, Immunohistochemistry

## Abstract

**Background:**

Hemoprotozoan diseases, especially trypanosomosis and theileriosis, adversely affect the productivity, growth, and performance of camels. Regular sampling and investigation of camels are challenging due to several factors. Consequently, there is a lack of knowledge on camel parasite genotyping, cytokine production, and oxidative stress parameters during infection.

**Methods:**

The present study investigated two critical blood protozoa infecting camels in Egypt, *Trypanosoma evansi* and *Theileria annulata*, using molecular methods, specifically 18S rRNA gene analysis. Following molecular confirmation, experimental infections were induced in Swiss albino mice to assess the expression of immune response genes and oxidative stress parameters. The study further explored the correlation between histopathological alterations and inflammatory reactions in the kidney, spleen, and liver of infected mice, alongside the immunohistochemical expression of caspase-3, proliferating cell nuclear antigen (PCNA), and tumor necrosis factor (TNF).

**Results:**

*Trypanosoma evansi* and *T. annulata* isolated from naturally infected camels were molecularly identified and deposited in GenBank under accession numbers OR116429 and OR103130, respectively. Infection with *T. evansi* and *T. annulata* caused significant adverse effects on the immune condition of infected mice, increasing the pathogenicity of the infection. This was evidenced by a significant increase in oxidative stress parameter levels in both naturally infected camels and experimentally infected mice compared to healthy controls. Furthermore, the expression of immune response genes was significantly elevated in infected mice. Immunohistochemistry analysis showed a pronounced upregulation of caspase-3, PCNA, and TNF in the infected groups relative to the control group. These findings are the first to be reported in Egypt.

**Conclusions:**

This study successfully identified and genotyped two economically important blood protozoa, *T. evansi* and *T. annulata*, from camels in Egypt. Additionally, the experimental animal model provided valuable insights into the immune response, oxidative stress, and histopathological changes induced by these parasites, demonstrating comparable results to naturally infected camels. These findings highlight the potential of this model to study parasite–host interactions and immune responses, contributing to a better understanding of the pathogenic mechanisms of *T. evansi* and *T. annulata* infections. This model may be useful for future studies focused on disease control and therapeutic interventions.

**Graphical Abstract:**

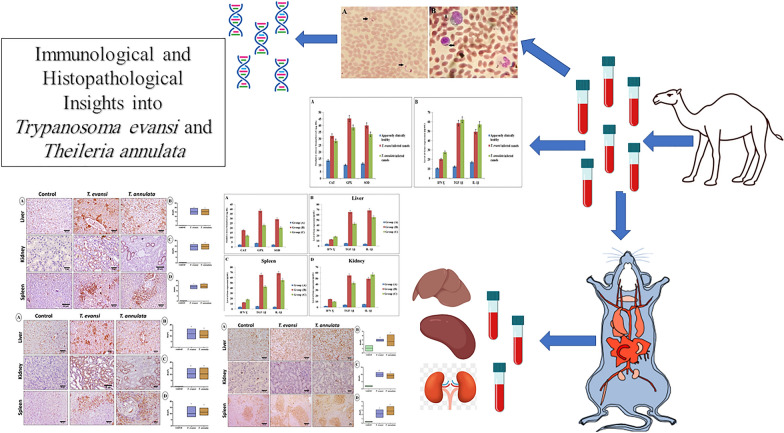

**Supplementary Information:**

The online version contains supplementary material available at 10.1186/s13071-024-06564-3.

## Background

Camels can survive under the harsh climatic conditions of the desert and contribute significantly to the socioeconomic uplift of a country, both as draft animals and as a protein source, as well as for various basic livelihood demands such as milk, meat, racing, riding, and packing [1]. Although camels are known to adapt to adverse desert climates, they may suffer from numerous parasitic infections, which severely limit their health progress [2]. Hemoprotozoan diseases such as anaplasmosis, babesiosis, trypanosomiasis, and theileriosis adversely affect infected camels, causing massive economic losses by affecting the quality of milk, meat, and other animal byproducts [3]. In Egypt, the two main vector-borne hemoprotozoans that typically manifest as chronic diseases in camels are tropical theileriosis (caused by *Theileria annulata*) and surra (caused by *Trypanosoma evansi*). The proportion of *T. evansi* in Egyptian camels ranges from 22.7% [4] to 50.51% [5]. *T. evansi* infections lead to significant productivity losses and can be lethal if not quickly identified and treated [6].

In Egypt, *T. annulata* is responsible for the subclinical form of the infection, but it causes significant alterations in blood and lipid profiles [1]. *T. annulata* has been reported to infect 21.1% of camels [7], rising to 38% in some cases [8]. Hemoprotozoan diseases may drastically impair the normal functioning of vital body organs [9]. Infection by *T. evansi* and *T. annulata* results in significant financial losses due to the severe clinical disease in infected and carrier camels [10]. Camels naturally tolerate a certain level of parasitemia, particularly in chronic cases, and the parasite may disappear from the blood while being stored in the bone marrow or other hemopoietic organs of the infected camel. Parasitological techniques, including direct microscopic observation of parasites in stained fixed blood films, are considered easy and inexpensive, but lack sensitivity [11]. However, during the acute stage of illness, when a high parasite counts occur, microscopic diagnosis is more reliable [12]. Serological methods lack sensitivity and specificity, as they rely on detecting parasite antibodies or antigens in animal blood [13, 14]. Among these techniques, diagnosis through the detection of specific parasite DNA using polymerase chain reaction (PCR) is considered highly useful for early diagnosis, even during the prepatent period or chronic late phase of infection [15]. Factors such as the amount and quality of DNA in the samples must be considered to maximize the effectiveness of PCR diagnosis [6].

Parasitic infections can induce oxidative stress through the production of reactive oxygen species (ROS) by the host, primarily as a defense against pathogen invasion [16]. The host’s antioxidant defense systems are sometimes activated, but oxidative stress occurs when ROS levels overwhelm these systems [17]. Excessive ROS production leads to oxidative damage to key cellular components—DNA, lipids, and proteins [18]. ROS-induced lipid peroxidation compromises cell membrane integrity, while oxidative DNA damage can result in mutations, replication errors, and cell death. Oxidative protein damage impairs cellular functions [19]. In the case of theileriosis, studies have demonstrated reduced antioxidant levels and elevated oxidative stress markers in affected animals [20].

A cell-mediated immune response is the most significant factor in diagnosing intracellular parasites, as it prevents their proliferation within the host and may trigger disease. Interferon-gamma (IFNγ) is essential for inducing host immunity and enhancing parasiticidal activity. In addition to oxidative stress, the immune response plays a critical role in controlling intracellular parasites. A robust cell-mediated immune response is essential to prevent parasite proliferation and subsequent disease. Key cytokines, such as IFNγ, enhance host immunity by promoting parasiticidal activity, while interleukin-1 beta (IL-1β) has been shown to inhibit parasite replication in vitro [21]. On the other hand, transforming growth factor 1 beta (TGF-1β), a regulatory cytokine, is less effective in treating acute trypanosomiasis, as demonstrated by [22]. TGF-1β, produced by various immune cells, activates immune responses following microbial invasion and induces cytotoxicity against tumor cells [23, 24].

Sampling and investigating camels poses significant challenges due to the nature of camel farming practices, which complicate rearing, handling, and securing methods [25]. Additionally, camels are predominantly distributed in tropical, developing regions, contributing to the limited understanding of camel parasite genotyping, cytokine production, and oxidative stress parameters during infection. This gap in knowledge underscores the need for studies like the present one. Therefore, the present study aimed to use molecular methods to identify two crucial blood protozoa infecting camels in Egypt (*T. evansi* and *T. annulata*). These parasites were subsequently used to induce experimental infections in mice. The study evaluated the expression of immune response genes (IFNγ, TGF-1β, and IL-1β) and oxidative stress parameters [superoxide dismutase (SOD), glutathione peroxidase (GPX), and catalase (CAT)] in both naturally infected camels and experimentally infected mice. Furthermore, the correlation between histopathological alterations and inflammatory reactions in the liver, spleen, and kidney, along with the immunohistochemical expression of caspase-3, proliferating cell nuclear antigen (PCNA), and TNF, was investigated to assess the impact of these infections.

## Methods

### Selection of *Trypanosoma *and *Theileria-*infected blood samples

From April to September 2023, 190 fresh noncoagulated blood samples were collected in sterile dipotassium EDTA-coated vacutainer tubes from the jugular veins of one-humped camels (*Camelus dromedarius*) directly before slaughter at El-Basatin Abattoir, Cairo, Egypt [25]. The samples were transported to the Parasitology Laboratory, Faculty of Veterinary Medicine, Cairo University, in an icebox. Each sample was identified and divided into two parts, one for molecular identification and the other for experimental infection. Giemsa-stained thin blood films were prepared and examined using a light microscope for infection levels. Blood samples with considerable parasitemia from *Trypanosoma* or *Theileria*, without other parasitic infections, were inoculated into mice as an animal model to meet the study’s objectives.

### Stained thin blood film preparation

Giemsa-stained thin blood films were prepared from each noncoagulated camel blood sample using the slide-to-slide method [26]. *Trypanosoma* infection between red blood cells (RBCs) and *Theileria* spp. gametocytes in RBCs or their schizonts inside circulating lymphocytes were diagnosed using an oil immersion lens (×1000). The level of parasitemia was calculated after investigating ten separate fields per sample. The parasites were identified according to previous methods [27].

### Molecular identification of the diagnosed parasites

#### DNA extraction, PCR, and sequencing

Phylogenetic analysis achieved an accurate identification of *Trypanosoma* and *Theileria* species in infected camel blood samples. Genomic DNA was extracted using the QIAamp DNA micro kit (Qiagen, USA), following the manufacturer’s instructions [28]. Samples from infected camel blood were genotyped using multiplex PCR (mPCR) against reference samples at the Central Laboratory for Evaluation of Veterinary Biologics, Agriculture Research Centre, Cairo, Egypt, following previous methods [29, 30].

According to previous studies [31, 32], the following primer sets were used for mPCR: *T. annulata*: 5′ ACTTTGGCCGTAATGTTAAAC 3′; 5′ CTCTGGACCAACTGTTTGG 3′; and *T. evansi*: 5′ TGCTTGTTTCAAGGACTTAGCCA 3′; 5′ CGCTGACTGAGAGAATCACGGTT 3′. The reaction was performed using Emerald Amp GT PCR master mix (Takara, Japan) in a 25 µL reaction mixture, which included 10 pmol of each primer (Metabion, Germany) and 5 µL of sample DNA as a template. The thermal profile included 30 cycles of denaturation (95 °C for 50 s), primer annealing (50 °C for 50 s), and extension (65 °C for 1 min), and a final extension of 10 min at 72 °C. The PCR product was electrophoresed on a 1.5% agarose gel with 10 µL/mL SYBER Safe (Thermo Scientific) in Tris–acetate EDTA buffer at 100 V for 45 min and photographed under ultraviolet transilluminators (ImageQuantLaz4000, GE Healthcare Life Science, Hammersmith, UK) [32].

Positive samples were sequenced for the small subunit ribosomal RNA gene (18S rRNA) at Macrogen Europe (The Netherlands), and the output was compared to sequences in GenBank using BLAST (http://blast.ncbi.nlm.nih.gov/) [33].

### The tested animal models

#### Experimental animals

The study utilized 90 inbred Swiss albino mice obtained from the Department of Animal and Poultry Management and Behavior, Faculty of Veterinary Medicine, Cairo University. The mice were between 6 and 8 weeks old, weighing between 26 and 32 g. They were housed in separate cages with air conditioning and provided unlimited access to filtered water and pellet rodent feed [34]. Humidity and room temperature were maintained at 50–70% and 25 ± 2 °C, respectively. During the 15 day acclimatization period, the animals appeared healthy, with no blood or enteric parasites detected through blood smears or copro-parasitological investigation [35].

### Animal infection

Ninety Swiss albino mice were divided into three equal groups (A, B, and C). Group A served as the control group, while group B was inoculated with *T. evansi*-infected camel blood, and group C was inoculated with *T. annulata*-infected camel blood. Each mouse in the control group (A) received sterile PBS intraperitoneally. According to a previous study [36], 30 mice per isolate (groups B and C) were injected with infected blood having *T. evansi* (5 × 10^5^ trypanosomes/mL) or *T. annulata* (1 × 10^7^ *Theileria*-infected RBCs) at a dose of 0.5 mL intraperitoneally to assess immunological, hematological, and histopathological changes induced in mice tissue due to infection with *T. evansi* or *T. annulata*. Daily blood examinations were performed to monitor infection status. Parasitemia was checked daily until infection was confirmed, and the overall death rate was recorded until day 30 postinfection (PI). Every 5 days, a blood film from the tail vein of five different mice from groups B and C was stained with Giemsa to determine parasite count. All animals were sacrificed on day 30 PI for histopathological analysis.

### Oxidative stress markers

Camel and mice blood were collected in tubes containing 0.5 mg/mL EDTA and stored at −20 °C until use. The blood samples were centrifuged for 10 min at 2000 rpm to estimate oxidative parameters [37]. The buffy coat and plasma were removed, and the erythrocyte pellet was packed and diluted in an EDTA–mercaptoethanol-stabilizing solution at a 1:9 (V/V) ratio. The pellet was stored at 4 °C for further analysis. The activity of SOD, GPX, and CAT was measured using these 10% packed erythrocytes. All oxidative parameter tests were completed within 2 hours of sample collection [38].

### Measurements of oxidative stress markers

Oxidative stress markers were assessed using specialized kits to measure SOD, GPX, and CAT activity in both positive and negative blood samples [39].

### Assessment of cytokine expression

#### RNA extraction

Total RNA was extracted from the blood of naturally infected camels and the liver, kidney, and spleen of experimentally infected mice with *Trypanosoma* and *Theileria* using 8.5 µL of sterile TRIzol reagent (Invitrogen Life Technologies, Carlsbad) and 10 pmol of Metabion (International AG). Two microliters of template DNA were used, following the manufacturer’s instructions. The RNA was diluted in RNase-free water and stored at −80 °C for gene expression analysis. The concentration and purity of RNA were determined using a Nano-Drop ND-1000 spectrophotometer (Nano-Drop Technologies Inc, Delaware, USA) [40].

### Real-time PCR (RT–PCR)

Cytokine genes IFNγ, IL-1β, and TGF-1β were quantified using real-time PCR. Beta-actin served as a reference gene for normalization. The primers listed in Table 1 were used for real-time RT–PCR using the Cepheid SmartCycler II (Sunnyvale, CA, USA). Samples were tested using SYBR Green PCR master mix (Applied Biosystems, USA), 0.5 µL of each primer (10 pmol), 1 µL of cDNA (400 ng), and 10.5 µL of RNase-free water. Positive and negative controls were included for each gene of interest. For β-actin, IFNγ, TGF-1β, and IL-1β genes, amplification cycles included an initial incubation at 95 °C for 5 min, followed by 40 cycles of denaturation at 95 °C for 30 s, annealing at 60 °C for 30 s, and extension at 72 °C for 30 s [41].

**Table 1:** Oligonucleotide primer used in the real-time PCR assay (F: forward, R: reverse)

Gene	Primer sequence	Reference
IFNγ	F: 5-AGC CAA ATT GTC TCC TTC TAC TTC-3 R: 5-CTG ACT TCT CTT CCG CTT TCT G-3	[22]
TGF-1 beta	F: 5-GGA CCT GGG CTG GAA GTG-3 R: 5-CTG CTC CAC CTT GGG CTT-3	[42]
IL-1 beta	F: 5-ATC TTC GAA ACG TCC TCC GAC-3 R: 5-CCT CT CCT TGC ACA AAG CTCA-3	[43]
β-Actin	F: 5-AAGAGAGGCATCCTGACCC-3 R: 5-TGTCACGGACGATTTCCGC-3	[44]

### Histopathology and immunohistochemistry

Formalin-fixed tissues from the liver, spleen, and kidney of experimentally infected mice were trimmed, embedded in paraffin, and stained with hematoxylin and eosin (H&E) as described previously [45]. Tissues were examined under a light microscope (Olympus, BX43) connected to a digital camera (DP27) and Cell Sens Dimensions software. Degeneration, necrosis, and inflammation were scored as follows: 0 = Normal, 1 =  < 25%, 2 = 25–50%, 3 = 50–75%, and 4 =  > 75% [46, 47]. The total lesion score for each mouse was calculated based on six fields at 200× magnification. Following a standardized protocol, immunohistochemical staining for caspase-3, PCNA, and TNFα was performed using the avidin–biotin–peroxidase complex method [48]. The area percentage of positive expression was calculated using ImageJ software (six fields/mouse/200×).

### Statistical analysis

Data were expressed as mean ± standard error. Statistical analyses were performed using SPSS version 28 (SPSS Inc., Chicago, IL, USA) [49]. A *t*-test for independent samples was applied, and a *P*-value of ≤ 0.05 was considered statistically significant [50].

## Results

### Microscopic examination of blood smears

Microscopic examination of Giemsa-stained thin blood films from all investigated animals revealed infection with *T. evansi* between the RBCs. The parasite exhibited a typical trypanosome spindle shape, with a central nucleus, subterminal kinetoplast, long free flagellum, and well-developed undulating membrane. The parasite was diagnosed in 33 samples (17.37%), with mixed infections of *Anaplasma* and *Theileria* identified in 14 samples (7.37%). Additionally, 22 samples (11.58%) were infected with *Theileria* spp., as its schizont was observed in circulating lymphocytes, displaying a spherical body with chromatin granules and blue cytoplasm adjacent to the kidney-shaped lymphocyte nucleus. Comma-shaped gametocytes were detected in some RBCs.

### Genotypic detection of *T. evansi* and *T. annulata*

The 18S rRNA gene was successfully amplified in all positive camel blood samples. The amplified products of 18S rRNA were 340 bp and 315 bp for *T. evansi* and *T. annulata*, respectively. BLAST analysis of the sequences confirmed the absolute similarity among all tested samples, identifying them as *T. evansi* and *T. annulata*. The sequences were submitted to GenBank, with accession numbers OR116429 and OR103130 for *T. evansi* and *T. annulata*, respectively. The 18S rRNA sequence of *T. evansi* showed 100% nucleotide similarity with accession number MT490639 and 99.17% similarity with MF737081 and LC546902. In the case of *T. annulata*, the 18S rRNA sequence displayed 100% nucleotide similarity with MT341857, MK415058, and AY524666.

### Oxidative stress and cytokine gene expression in both naturally infected camels and experimentally infected mice with *T. evansi* and *T. annulata*

#### In naturally infected camels

**Fig. 1:**
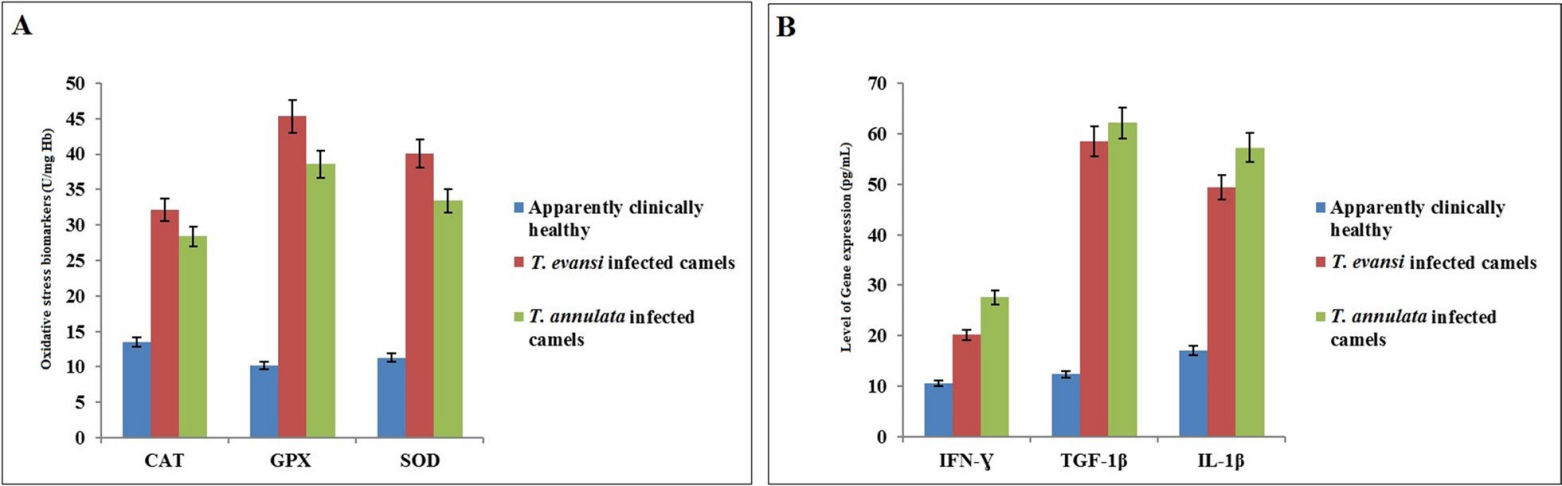
**A** Oxidative stress biomarkers (CAT, GPX, and SOD) and **B** cytokines expression (IFNγ, TGF-1β, and IL-1β) in the blood of apparently healthy and naturally infected camels

The biochemical profile of antioxidant and inflammatory markers in naturally infected camels in comparison with their level in apparently healthy ones are shown in Fig. 1. There was also a significant increase (*P* ≤ 0.05) in the concentration of the investigated oxidative stress markers (CAT, SOD, and GPX) in naturally infected camels in comparison with the control noninfected camels. The elevation was higher in the case of infection with *T. evansi* than that recorded in camels infected by *T. annulata* (Fig. 1A). At the same time, there was a significant increase (*P* ≤ 0.05) in the level of immunogenic cytokines (IFNγ, TGF-1β and IL-1β) in *T. evansi* and *T. annulata* naturally infected camels in comparison with the control camels. The difference is considered to be higher in animals infected by *T. annulata* than those infected by *T. evansi* (Fig. 1B).

### Experimentally infected mice

**Fig. 2:**
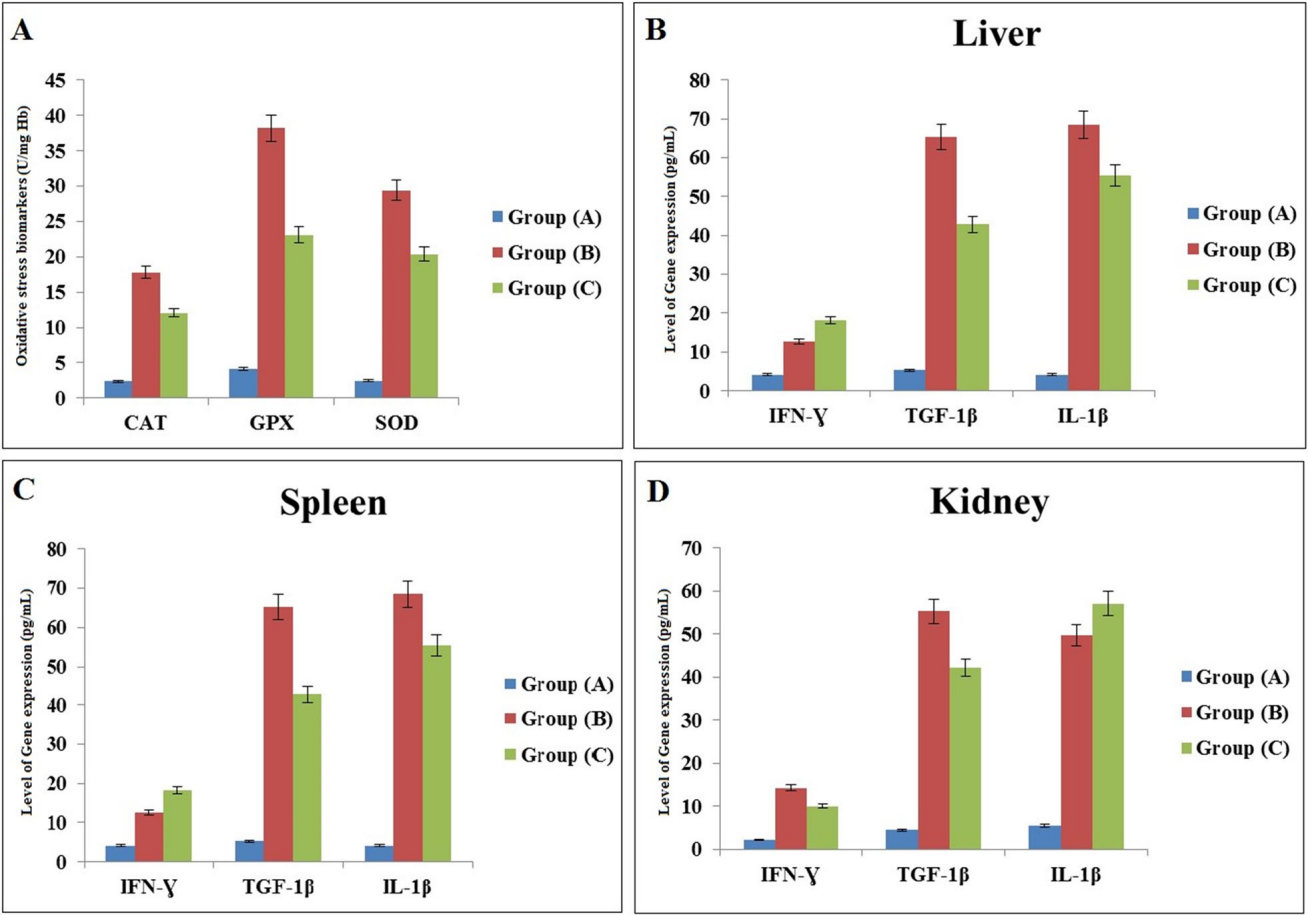
**A** Blood oxidative stress markers (CAT, GPX, and SOD), **B** cytokines expression (IFNγ, TGF-1β, and IL-1β) in the liver, **C** spleen, and **D** kidney of control and experimentally infected mice with *T. evansi* and *T. annulata*

After confirming parasitemia in inoculated mice, oxidative stress markers were assessed in each group. The results showed that levels of CAT, GPX, and SOD were higher in both *Trypanosoma*- and *Theileria*-infected groups compared to the healthy control group (Fig. 2A). Additionally, cytokine gene expression levels (IFNγ, TGF-1β, and IL-1β) were elevated in infected mice (Fig. 2B–D). The observed alterations in oxidative stress and gene expression parameters suggest a connection to the pathophysiology of mice infected with *T. evansi* and *T. annulata*.

### Histopathology and immunohistochemistry

**Fig. 3:**
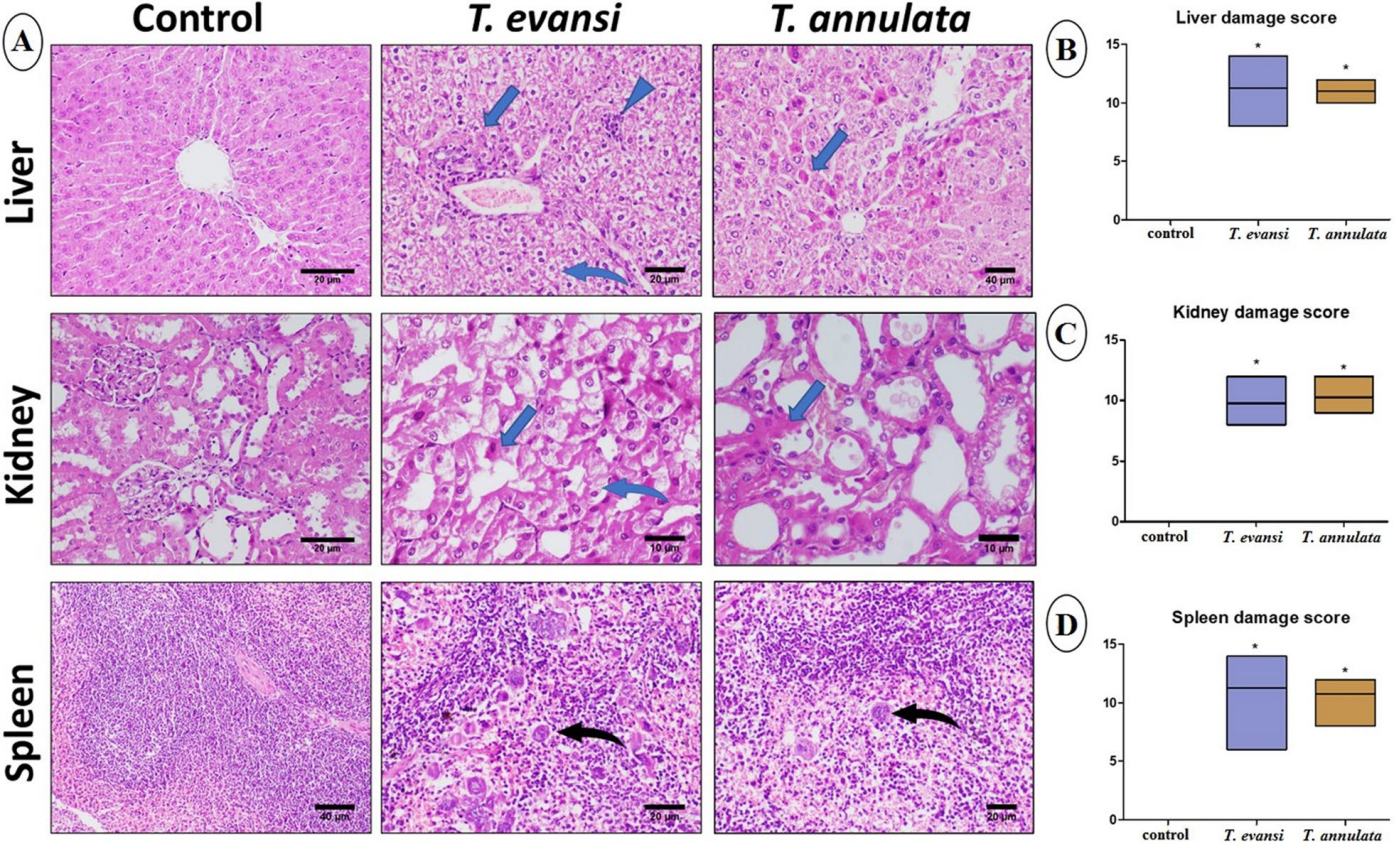
**A** Photomicrographs of liver, kidney, and spleen stained by H&E. **B**, **C**, and **D** Histological damage scores in liver, kidney, and spleen tissues, respectively. Data are presented as median ± SD, applying a *t*-test followed by the Mann–Whitney test (*P ≤ 0.05). Notable features include inflammatory cell infiltration (arrowhead), coagulative necrosis (blue arrow), vacuolation of hepatocellular cytoplasm (curved blue arrow), and extramedullary megakaryopoiesis (curved black arrow)

**Fig. 4:**
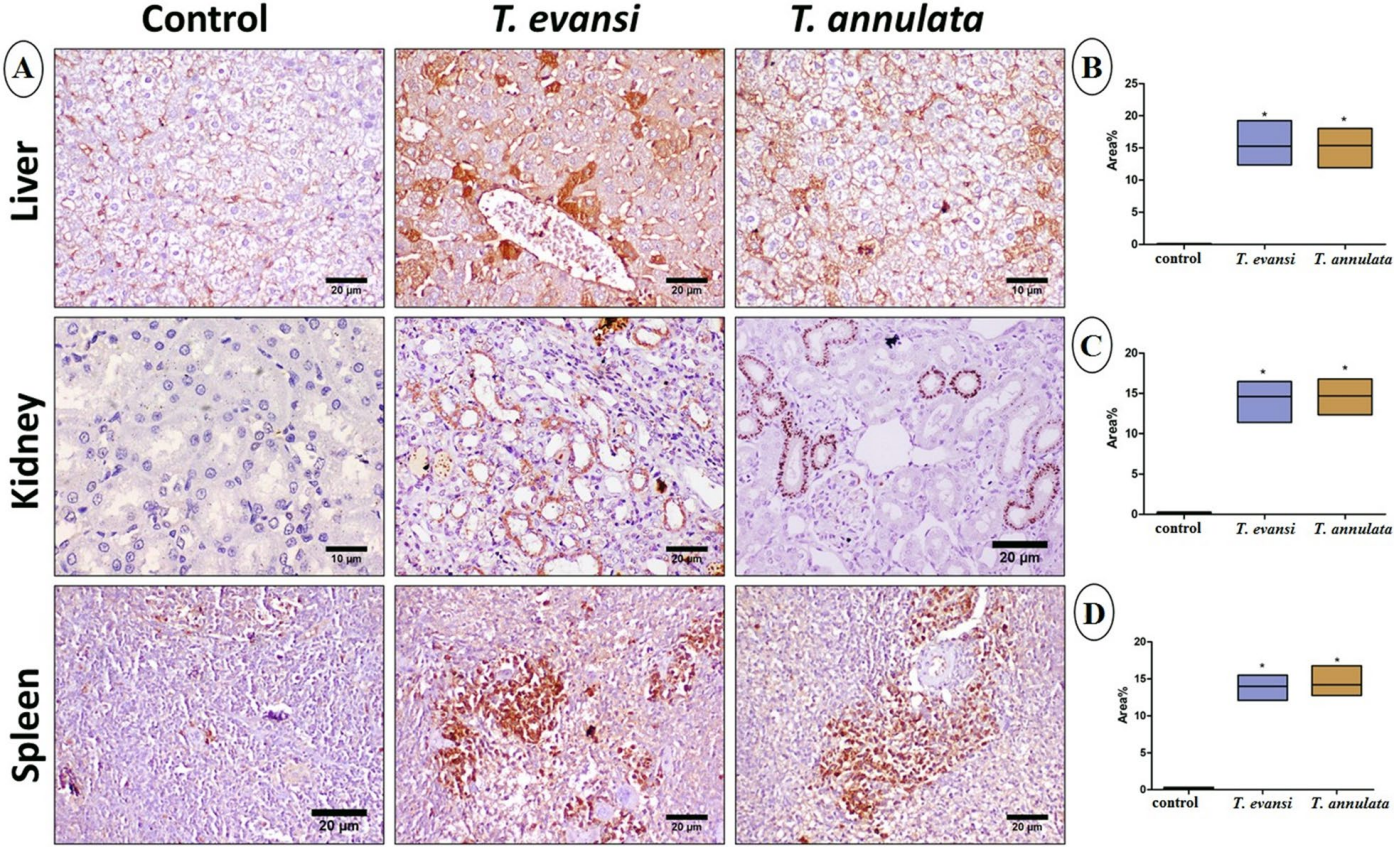
**A** Photomicrographs of liver, kidney, and spleen stained with caspase-3 immunohistochemistry. **B**, **C**, and **D** Immunohistochemical analysis of the area percentage of caspase-3 expression in the liver, kidney, and spleen, respectively. Data are expressed as mean ± SD (*P ≤ 0.05)

**Fig. 5:**
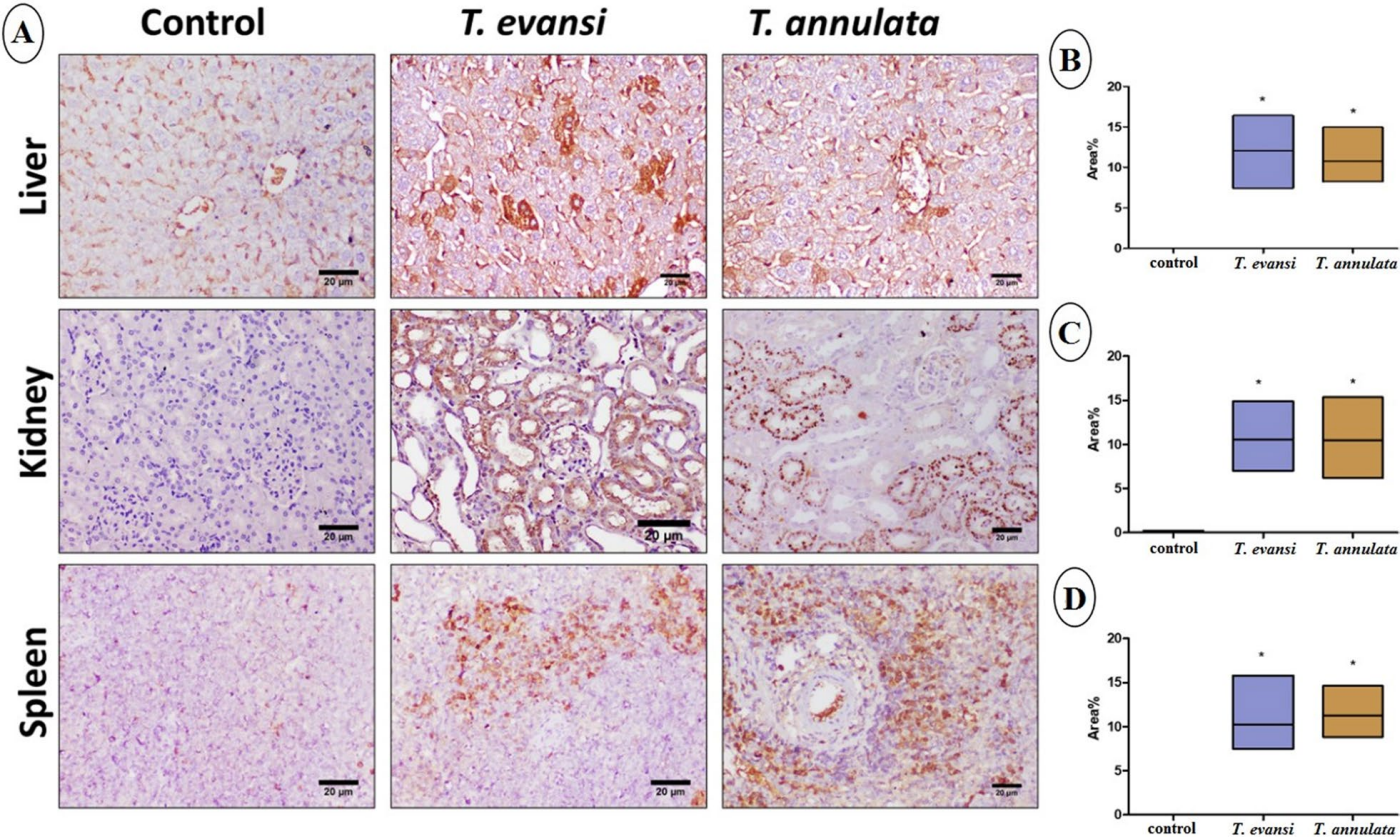
**A** Photomicrographs of liver, kidney, and spleen stained with TNF immunohistochemistry. **B**, **C**, and **D** Immunohistochemical analysis of the area percentage of TNF expression in the liver, kidney, and spleen, respectively. Data are expressed as mean ± SD (*P ≤ 0.05)

**Fig. 6:**
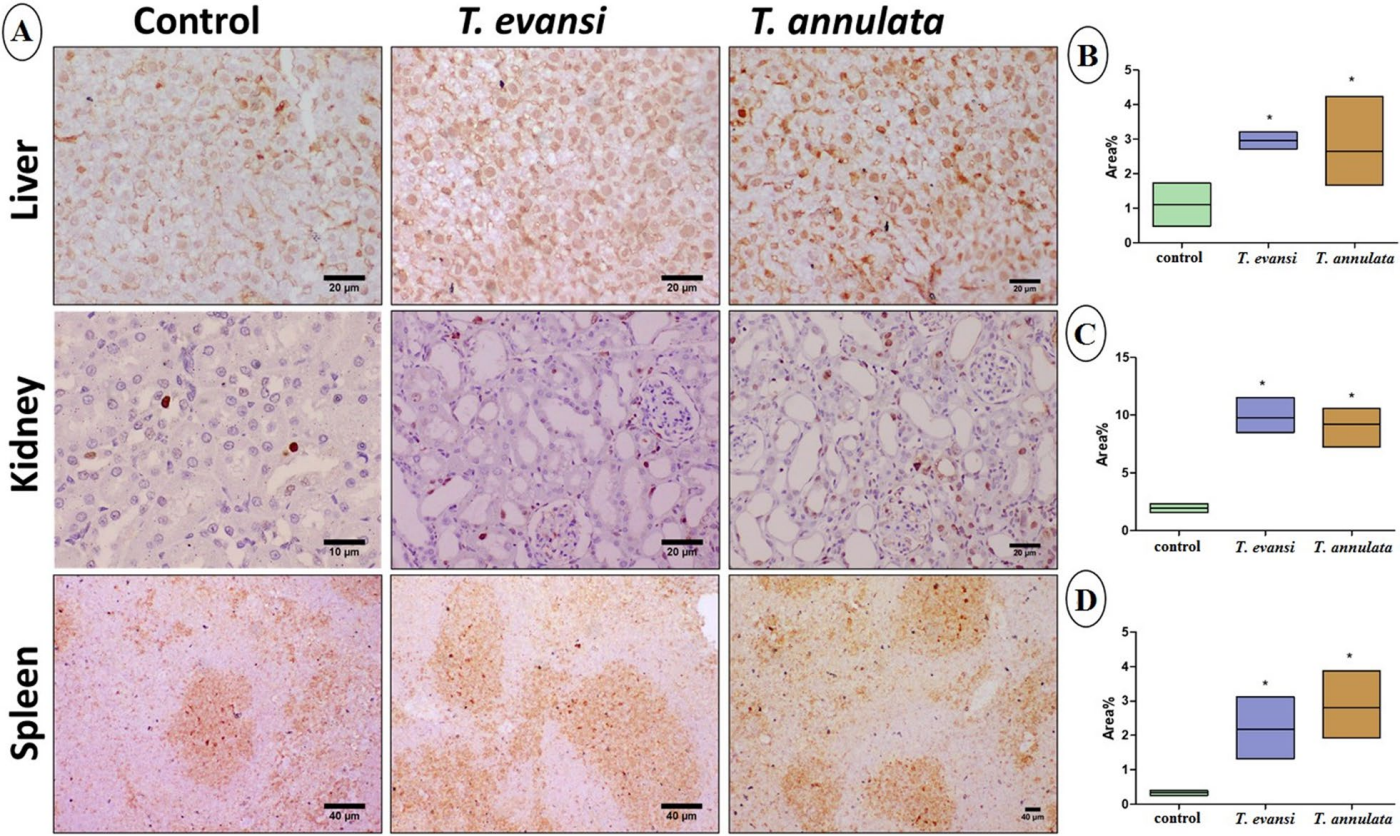
**A** Photomicrographs of liver, kidney, and spleen stained with PCNA immunohistochemistry. **B**, **C**, and **D** Immunohistochemical analysis of the area percentage of PCNA expression in the liver, kidney, and spleen, respectively. Data are expressed as mean ± SD (*P ≤ 0.05)

Microscopic examination of the control group showed normal liver, kidney, and spleen histological architecture, with mild splenic extramedullary hematopoiesis and megakaryocytes. In contrast, histopathological examination of the liver in *T. evansi*- and *T. annulata*-infected groups revealed significant alterations, including diffuse vacuolization of hepatocellular cytoplasm, hepatocellular necrosis, and mononuclear cell infiltration extending into portal areas. Examination of kidney tissues revealed necrobiotic changes in the renal tubular epithelium, while the spleen showed congestion, increased extramedullary hematopoiesis, megakaryopoiesis, and proliferation of erythroid elements (Fig. 3). Immunohistochemistry analysis demonstrated significant upregulation of caspase-3, PCNA, and TNF expression in hepatic, renal, and splenic tissues from infected groups compared to control mice (Figs. 4, 5, and 6).

## Discussion

*Camelus dromedarius* (one-humped camels) significantly contribute to the socioeconomic development of numerous countries. However, camel meat and interactions with camels pose substantial zoonotic disease risks due to their susceptibility to various infections [51, 52]. Hemoprotozoan diseases such as anaplasmosis, babesiosis, trypanosomiasis, and theileriosis negatively impact the productivity, growth, and performance of animals and humans [3]. These diseases cause massive economic losses by affecting the quality of milk, meat, and other animal byproducts [53].

Phylogenetic analyses and the taxonomic identification of *Theileria* spp. and trypanosomes depend on molecular analysis of ribosomal gene sequences [31]. The DNA sequence of the nuclear small subunit (18S) rRNA, has become the most widely accepted method for rapid diagnosis and classification of different protozoan parasites [54]. This study determined the phylogenies and relatedness of the Egyptian isolates using phylogenetic trees based on 18S rRNA analysis. The Egyptian isolates grouped with *T. evansi* and *T. annulata* obtained from GenBank based on their 18S rRNA sequences. Previous studies [30, 55, 56] that isolated *T. evansi* from Egypt, Saudi Arabia, and Paraguay, are consistent with the 18S region-based results. However, the *T. annulata* findings align with studies on isolates from China, Italy, and Turkey [29, 57, 58].

Concerning the effect of infection on variations in antioxidant and inflammatory markers in natural or experimentally infected animals, it is known that the immune system is stimulated by increasing exposure to infections, relative to the level of tissue damage that occurs as a result of this infection [21]. As the body’s army, the immune system keeps out bacterial and parasitic invaders. Its primary components are glycoproteins, which are small proinflammatory cytokines. They are effective in regulating the interactions and communication between different immune cells. In the current study, cytokine production was triggered by *Trypanosoma* and *Theileria* infection, initiating an immune response. Proinflammatory cytokines are produced by various immune cells in response to the presence, endurance, mobility, and proliferation of protozoa in an animal’s body, and the associated wounded tissues [21, 59].

Oxidative stress occurs when the body’s defense mechanisms against free radicals and ROS are outmatched. In the current study, infected camels had significantly higher levels of CAT, SOD, and GPX. The obtained results were in agreement with previous publications [60, 61]. Regarding the variation in the elevated levels of the estimated parameters related to infection with *T. evansi* or *T. annulata* in the naturally infected camels in this study, the authors attribute this to other factors such as the general health conditions and immune status of the animals, as well as the level of parasitemia. Furthermore, the possibility of other unapparent infections may also contribute to these variations. Lipid peroxidation and oxidative reactions play a role in the pathophysiology of anemia. According to our findings, CAT, GPX, and SOD levels were higher in experimentally infected mice compared to controls. These results align with previous studies [16, 62], which found increased CAT and SOD activity in the hearts of *T. evansi*-infected mice. In contrast to our results, a previous study [63] reported increased CAT activity and decreased SOD activity in sheep naturally infected with *T. annulata*, which may indicate weakened CAT antioxidant capacity. Numerous studies have reported that SOD demonstrates the highest catalytic efficiency and resistance to oxidative stress among known enzymes [64]. However, significant reductions in SOD activity have been observed in cattle affected by theileriosis [65]. Additionally, elevated GPX activity in the blood of rats infected with *T. evansi*has been observed [66]. Conversely, our findings contradict another study [67], which found a decrease in GPX activity in the whole blood of rats infected with trypanosomosis.

Cytokines play an essential role in regulating humoral and cellular immune responses. While cytokine expression, particularly during the subclinical stage, may provide insights into the immune response against *Trypanosoma* and *Theileria* infections, it may not be the most reliable standalone diagnostic tool due to variability in cytokine levels influenced by host factors and nonspecificity. Therefore, cytokine profiling is better used as a complementary approach alongside conventional diagnostic methods, such as PCR or serology, to enhance diagnostic accuracy. To the best of our knowledge, this is the first study in Egypt to address the gene expression of IFNγ, TGF-1β, and IL-1β in both naturally infected camels and experimentally infected mice with *T. evansi* and *T. annulata* as an anomalous model for mammals to simplify the diagnosis of intracellular parasites. According to our findings, levels of IFNγ, TGF-1β, and IL-1β in both naturally infected camels and experimentally infected mice were high compared to controls. These results align with previous studies, which reported that the inflammatory response and parasitemia occurring in infected animals may be linked to this increase [22, 68, 69]. IFNγ is believed to be the first inflammatory cytokine crucial in triggering macrophage activation when the parasite stimulates the cells with antigen. Activated macrophages induce TGF-1β and IL-1β [70, 71]. Conversely, earlier research on intracellular parasites revealed lower levels of IFNγ mRNA expression and higher levels of TGF-1β and IL-1β expression compared to healthy animals [72–74]. Additionally, IL-1β inhibits the in vitro proliferation of parasites. A positive correlation between TGF-1β and IL-1β has been observed, indicating that IL-1β may be upregulated in response to TGF-1β upregulation [75]. This increase in pathogenicity and immune suppression exacerbates the overall health of infected camels, reducing productivity and increasing susceptibility to other pathogens.

Histopathological examination revealed significant damage to the liver, kidney, and spleen of the infected group, attributed to ROS production and hypersensitivity to infective agents [76]. Furthermore, parasitic toxin release causes degeneration and necrosis of tissues [77]. Immunohistochemical analysis showed increased caspase-3 upregulation in the infected groups compared to controls, indicating its involvement in apoptosis. This finding explains the degeneration and apoptosis observed in the kidney, liver, and spleen on histopathological analysis. Cellular apoptosis in infected groups may result from toxic byproducts inducing hypoxia and inflammation [73]. TNF expression was elevated in the liver, kidney, and spleen of the infected groups. *T. evansi* and *T. annulata* have been reported to induce macrophage activation, which stimulates multiple inflammatory cytokines such as TNF, IL-2, IL-4, IL-6, and IL-12 [72]. PCNA, a cell proliferation marker, is typically expressed in hepatocytes, renal tubular cells, and splenocytes during regeneration [78]. The increased PCNA expression in the infected mice tissues indicates regeneration initiation. Moreover, a positive correlation was found between apoptotic markers such as BAX, caspase-3, and PCNA, as previously reported [79].

## Conclusions

This study effectively identified and genotyped two economically important blood protozoa, *T. evansi* and *T. annulata*, from camels in Egypt, and the relevant genomes were deposited in GenBank. Furthermore, the experimental animal model gave valuable insights into the immunological response, oxidative stress, and histopathological alterations caused by these parasites, with results comparable to naturally infected camels. These findings emphasize the model’s potential for studying parasite–host interactions and immune responses, which will help us understand the pathogenic mechanisms of *T. evansi* and *T. annulata* infections. This model could be beneficial in future studies on disease control and treatment interventions.

## Supplementary Information

Supplementary Material 1.

## Data Availability

No datasets were generated or analysed during the current study.

## Abbreviations

PCNA: Proliferating cell nuclear antigen
TNF: Tumor necrosis factor
GPX: Glutathione peroxidase
CAT: Catalase
SOD: Superoxide dismutase
IFN-γ: Interferon-gamma
TGF-1β: Transforming growth factor-1 beta
IL-1β: Interleukin-1 beta
*T. annulata*: *Theileria annulata*
*T. evansi*: *Trypanosoma evansi*
H&E: Hematoxylin and eosin
