# Supplementary material for: Novel insights into antioxidant status, gene expression, and immunohistochemistry in an animal model infected with camel-derived Trypanosoma evansi and Theileria annulata
Source: Parasit Vectors. 2024 Nov 18;17:474. doi: 10.1186/s13071-024-06564-3 (PMC11575088; doi:10.1186/s13071-024-06564-3)
Supplement: Supplementary file 1 — Supplementary Material 1. [file 13071_2024_6564_MOESM1_ESM.doc]

The figure showed (A) *T. evansi* in blood smears. (B) *Theileria* spp. Schizont in lymphocyte (Fixed blood film stained with Giemsa-under oil immersion lens X1000 magnification).
